# Data on sorption of organic compounds by aged polystyrene microplastic particles

**DOI:** 10.1016/j.dib.2018.03.053

**Published:** 2018-03-16

**Authors:** Thorsten Hüffer, Anne-Katrin Weniger, Thilo Hofmann

**Affiliations:** University of Vienna, Department of Environmental Geosciences and Environmental Science Research Network, Althanstrasse 14, 1090 Vienna, Austria

## Abstract

This article contains data on experimental sorption isotherms of 21 probe sorbates by aged polystyrene microplastics. The polymeric particles were subjected to an UV-induced photo-oxidation procedure using hydrogen peroxide in a custom-made aging chamber. Sorption data were obtained for aged particles. The experimental sorption data was modelled using both single- and poly-parameter linear free-energy relationships. For discussion and interpretation of the presented data, refer to the research article entitled “Sorption of organic compounds by aged polystyrene microplastic particles” (Hüffer et al., 2018) [1].

**Specifications Table**

**Table:** 

Subject area	*Chemistry*
More specific subject area	*Environmental Chemistry*
Type of data	*Tables, figures*
How data was acquired	*GC–MS (Agilent 7890A gas chromatograph coupled to a 5975C mass spectrometer equipped with ITEX2 option for CombiPal autosampler from Axel Semrau, Sprockhövel, Germany), Sigma Plot 12.0 (Windows) for model fits and statistical analyses*
Data format	*Analyzed data*
Experimental factors	*Polystyrene microplastics were exposed to an UV-induced photo-oxidation procedure with H*_*2*_*O*_*2*_
Experimental features	*Sorption isotherms of 21 probe sorbates were performed using UV-aged polystyrene microplastics as sorbent*
Data source location	*Vienna, Austria*
Data accessibility	*The data are available within this article*

**Value of the data**

● Sorption isotherm data for UV-aged polystyrene microplastic were determined for 21 molecular probe sorbates covering a broad spectrum of molecular substance classes.
● Modelling data provided information for the interpretation of molecular interactions between UV-aged polystyrene microplastics and organic compounds.
● Modelling data are valuable for the prediction of sorption by UV-aged polystyrene microplastics and allow a comparison with data from other aging processes and environmentally relevant polymers particles.

## 1 Data

**Fig. 1:**
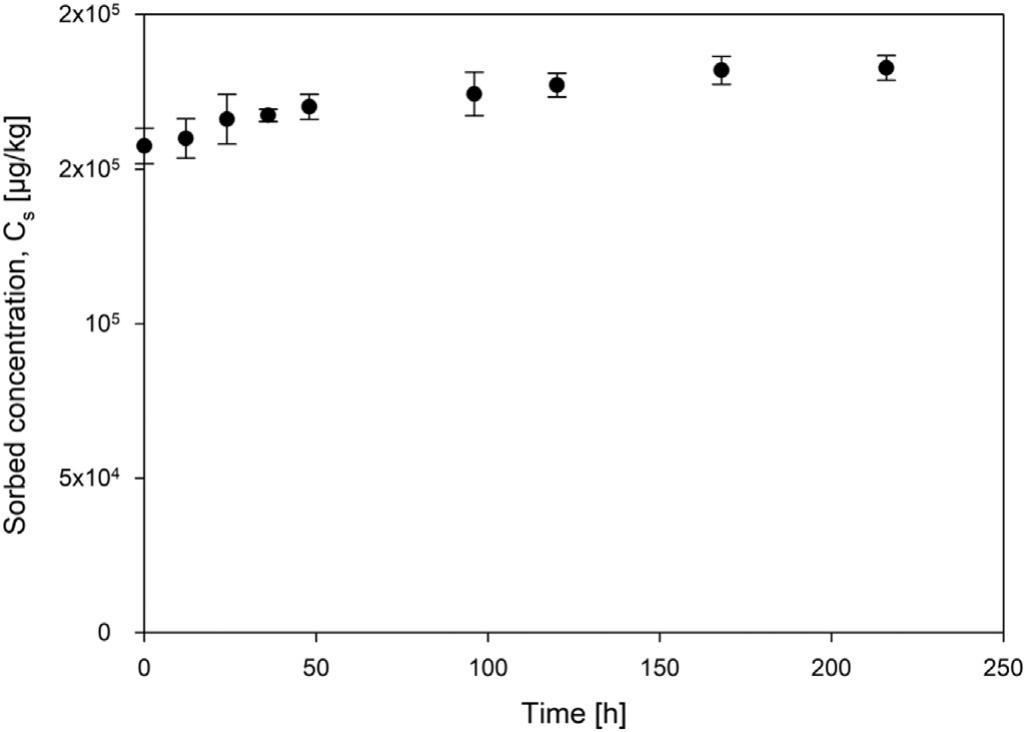
Sorption kinetics of naphthalene by aged polystyrene microplastics.

**Fig. 2:**
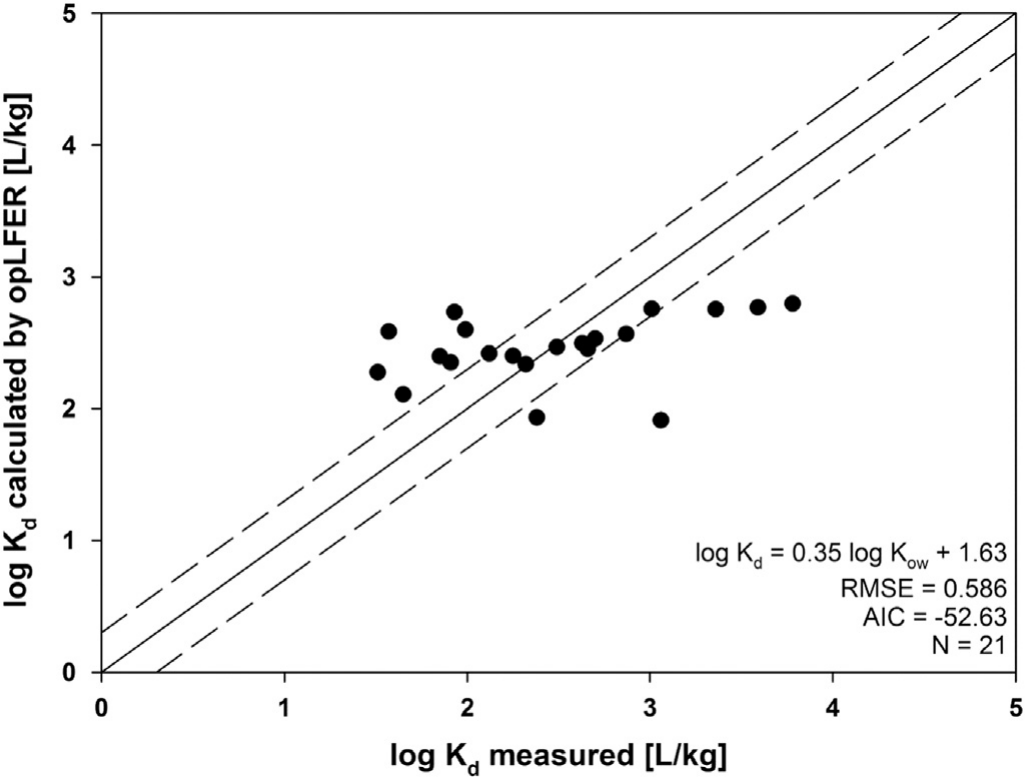
Comparison between experimentally determined log Kd and calculated by opLFER using log *K_ow_*.; AIC: Akaike's Information Criterion; RMSE: root mean squared error; *N*: number of data points.

Physico-chemical properties of the probe sorbates are given in Table 1. Fig. 1 shows sorption kinetics data of naphthalene by aged polystyrene microplastics (PSMP). Freundlich model fit data from sorption isotherms are shown in Table 2. A comparison of Freundlich fit model data between pristine and UV-aged polystyrene microplastics is given in Table 3. Data from statistical analyses of poly-parameter linear free-energy relationship model are shown in Table 4, Table 5, Table 6, Table 7. Single-parameter linear free-energy relationships for sorption of organic compounds by PS micro- and nanoplastics are given in Table 8. Fig. 2 visualizes the correlation between experimental distribution coefficients of probe sorbates by aged polystyrene microplastics and octanol-water partitioning coefficients.

**Table 1:** Probe sorbates, selected physico-chemical properties, and solute descriptors.^a^

**Compound**	**log S**_**w**_^b^	**log K**_**aw**_^c^	**log K**_**ow**_^d^	**E**	**S**	**A**	**B**	**V**	**L**
n-Hexane (nHex)	0.98	1.73	3.90	0.00	0.00	0.00	0.00	0.954	2.688
Isohexane (iHex)	1.15	1.75	3.21	0.00	0.00	0.00	0.00	0.954	2.503
Cyclohexane (cHex)	1.74	0.78	3.44	0.31	0.10	0.00	0.00	0.845	2.964
Dichloromethane (DCM)	4.11		1.15	0.39	0.57	0.10	0.05	0.494	2.019
Tetrachloromethane (TCE)	2.90	− 0.02	2.83	0.46	0.38	0.00	0.00	0.739	2.823
Di-n-propyl ether (DPE)	3.69	− 0.97	2.03	0.01	0.22	0.00	0.45	1.013	2.803
2-octanone (2ON)	2.95	− 1.98^a^	2.37	0.11	0.68	0.00	0.51	1.252	4.257
Hexanenitrile (HNT)	3.39	− 2.30^a^	1.66	0.17	0.90	0.00	0.36	0.968	3.513
1-nitrohexane (1NH)	2.26		2.70	0.20	0.95	0.00	0.29	1.128	4.416
2-octanol (2OL)	3.05		2.90	0.16	0.36	0.33	0.56	1.295	4.339
3-ethylhexanol-3 (3EH)	3.17		2.69	0.20	0.30	0.31	0.64	1.154	3.805
2,6-dimethylheptanol-2 (DMH)	2.76	− 2.30^a^	3.11	0.13	0.27	0.31	0.60	1.435	4.469
Benzene (BEZ)	3.25	− 0.65	2.17	0.61	0.52	0.00	0.14	0.716	2.786
Toluene (TOL)	2.72	− 0.60	2.69	0.60	0.52	0.00	0.14	0.857	3.325
Chlorobenzene (CBZ)	2.70	− 0.80	2.84	0.72	0.65	0.00	0.07	0.839	4.230
Naphthalene (NAP)	1.49		3.30	1.34	0.92	0.00	0.20	1.085	5.161
Benzothiazole (BTZ)	3.63		2.01	1.33	1.30	0.00	0.39	0.969	5.522
Ethylbenzoate (EBT)	2.86	− 2.38	2.67	0.69	0.85	0.00	0.46	1.214	5.075
4-nitrotoluol (4NT)	2.65	− 2.76	2.37	0.87	1.11	0.00	0.28	1.032	5.154
1-naphthol (1NP)	2.94		2.85	1.52	1.05	0.60	0.37	1.144	6.284
2-chlorophenol (2CP)	4.05	− 3.24	2.15	0.85	0.88	0.32	0.31	0.898	4.178

a solute descriptors were obtained from Ref. [2].

b Sw: aqueous solubility [mg L^−1^] at 25 °C from Ref. [3].

c *K_aw_*: air-water partitioning constant [–] from Ref. [4] or calculated using a combination of Eq. (6)–(15) and (6)–(17) from ref [4]: $K_{aw}=\frac{p_{i}[atm]}{C_{i,sat}[mol\;L^{-1}]}\cdot \frac{1}{T\;[K]\cdot R[atm\;L\;mol^{-1}\;K^{-1}]}$.

d *K_ow_*: octanol-water partitioning constant [–] from Ref. [3].

**Table 2:** Data of the Freundlich Model fit to the experimental sorption isotherms.

**Compound**	***K**_**F**_*	***n***	***R***^**2**^	***N***
nHex	1.19E+04 ± 7.87E02	0.89 ± 0.02	0.956	15
iHex	3.77E+03 ± 3.98E+02	1.11 ± 0.05	0.969	15
cHex	4.19E+02 ± 8.44E+01	1.17 ± 0.04	0.989	13
DCM	5.87E+01 ± 6.67E+00	0.92 ± 0.02	0.981	14
TCM	1.69E+02 ± 2.58E+01	1.10 ± 0.03	0.985	15
DPE	6.78E+01 ± 1.23E+01	1.01 ± 0.03	0.936	15
2ON	5.84E+01 ± 2.03E+01	1.07 ± 0.06	0.999	15
HXN	5.54E+01 ± 2.30E+01	0.90 ± 0.06	0.935	15
1NH	3.07E+02 ± 1.50E+02	0.92 ± 0.07	0.968	10
2OL	2.13E+02 ± 5.47E+01	0.92 ± 0.06	0.917	10
3EH	5.81E+01 ± 1.61E+01	0.80 ± 0.05	0.967	14
DMH	9.08E+01 ± 1.68E+01	0.81 ± 0.04	0.924	14
BEZ	2.54E+02 ± 5.25E+01	0.94 ± 0.03	0.977	15
TOL	3.37E+02 ± 7.68E+01	0.96 ± 0.03	0.920	15
CBZ	1.80E+03 ± 1.83E+02	0.83 ± 0.02	0.961	15
NAP	1.81E+03 ± 4.41E+02	1.02 ± 0.04	0.987	12
BTZ	2.65E+02 ± 6.42E+01	1.00 ± 0.04	0.968	12
EBT	1.20E+04 ± 3.50E+03	0.70 ± 0.04	0.954	11
4NT	3.15E+02 ± 1.70E+02	1.00 ± 0.07	0.980	11
1NT	6.72E+02 ± 2.21E+02	0.93 ± 0.05	0.952	9
2CP	3.78E+01 ± 1.37E+01	1.10 ± 0.05	0.946	14

*K_F_*: Freundlich coefficient; *n*: Freundlich exponent; *R*^2^ regression coefficient; *N*: number of data points.

**Table 3:** Comparison of Freundlich parameters obtained for pristine and aged polystyrene microplastic particles.

	Pristine PS [5]			Aged PS [1]		
Sorbate	*K_F_*	*n*	*R*^2^	*K_F_*	*n*	*R*^2^
nHex	14,643.2	0.762	0.941	11,906.5*	0.891**	0.911
cHex	2566.6	0.742	0.964	734.7**	0.999**	0.909
BEZ	800.3	0.844	0.981	265.5**	0.931**	0.920
CBZ	3421.1	0.810	0.971	1695.0**	0.902**	0.961
NAP	2333.3	0.906	0.936	1806.2*	0.999	0.917

* *p* < 0.05.

** *p* < 0.01.

## 2 Statistical analyses of ppLFER

See Table 4, Table 5, Table 6, Table 7, Table 8 and Fig. 2.

**Table 4:** Parameters for ppLFER using ESABV descriptors.

	**Coefficient**	**SE**	***p*-Value**
e	0.6708	0.1613	0.0008
s	− 0.7491	0.2012	0.0020
a	− 1.5278	0.4399	0.0034
b	− 3.5158	0.4110	< 0.0001
v	2.8607	0.3012	< 0.0001
c	0.7365	0.2510	0.0102

**Table 5:** ANOVA for ppLFER using ESABV descriptors.

	**df**	**SS**	**MS**	***F*-value**	***F*-critical**
Model	5	7.971	1.594	45.162	1.62E−08
Residue	15	0.5295	0.0353		
Total	20	8.500

**Table 6:** Parameters for ppLFER using SABVL descriptors.

	**Coefficient**	**SE**	***p*-Value**
s	− 0.10188	0.3155	0.0056
a	− 1.4273	0.4863	0.0102
b	− 3.6072	0.4556	< 0.0001
v	1.4481	0.5748	0.0236
l	0.4252	0.1279	0.0046
c	1.0089	0.2979	0.0041

**Table 7:** ANOVA for ppLFER using SABVL descriptors.

	**df**	**SS**	**MS**	***F*-value**	***F*-critical**
Model	5	7.844	1.569	35.841	7,97E−08
Residue	15	0.6565	0.0437		
Total	20	8.5000			

All parameters were calculated at a 95% confidence level.

SE: standard error of estimates.

df: degrees of freedom.

SS: sum of squares.

MS: mean square.

**Table 8:** opLFER parameters for sorption organic compounds by polystyrene micro- and nanoplastics.

**Sorbent**	**Sorbates**	**opLFER**	**AIC**	**RMSE**	***N***
Aged PS microplastics [1]	Non-ionic organics	Log *K_d_* = 0.35 ± 0.09 log *K_ow_* + 1.63 ± 0.24	− 52.63	0.586	21
Pristine PS microplastics [5]	Non-polar organics	Log *K_d_* = 0.92 log K_ow_ + 0.31	− 24.85	0.219	7
Surface coated PS nanoplastics [6]	PCBs	Log *K_d_* = 1.01 log *K_ow_* + 0.36	− 69.46	0.566	17
Surface coated PS nanoplastics [7]	PAH	Log *K_d_* = 0.65 log *K_ow_* + 3.87	− 38.39	0.131	6

AIC: Akaike's Information Criterion; RMSE: root mean squared error; *N*: number of data points.

## 3 Experimental design, materials and methods

### 3.1 Materials

Polystyrene microplastics were purchased as a powder from Goodfellow Cambridge Ltd. (Huntingdon, UK.). The particles were sieved to a size fraction between 125 and 250 µm. The sorbates included apolar aliphatics, monopolar aliphatics, bipolar aliphatics, non-polar aromatics, monopolar aromatics, and bipolar aromatics (Table 1).

### 3.2 Aging of polystyrene microplastic particles

A custom-made aging chamber was used for particle aging. The particles were weighed into quartz glass petri dishes containing 50 mL of H_2_O_2_ (10 vol%). The samples were then irradiated for 96 hours using UV light (4*15 W UVC-bulbs, max. wavelength at 254 nm). The aged particles were washed with deionized water and dried prior to the sorption batch experiments.

### 3.3 Sorption experiments

20–60 mg of the sorbent particles were weighed into 20-mL amber headspace screw vials. 10 mL of 0.01 M CaCl_2_ was added as background solution. The vials were closed with screw caps with butyl/PTFE-lined septa and wrapped in aluminum foil. After shaking overnight at 125 rpm to pre-wet the sorbent, the samples were spiked with sorbate standard solutions (methanol did not exceed 0.5%, to avoid co-solvent effects). The vials were then shaken for 7 days at 125 rpm for equilibration at a temperature of 25 ± 2 °C. Equilibration was determined using naphthalene as a probe sorbate (Fig. 1). The vials were then placed on the tray of the autosampler at least 2 hours prior to analysis. The concentrations in the head space of the vials was measured with a GC–MS-system either using in-tube microextraction or direct injection of 500 µL of the headspace sample. The sorbed concentrations were calculated using a mass balance and the air-water partitioning constants of the sorbates (Table 1).

### 3.4 Data analysis

Distribution coefficients between the aqueous phase and the sorbent (*K_d_*) [L/kg] were calculated for all sorbates at a constant sorbate loading on aged PSMP of 1000 µg/kg, using the Freundlich equation:

(1) $$K_{d}=\frac{C_{s}}{C_{w}}=K_{F}C_{w}^{n-1}$$

where *C_s_* [μg/kg] and *C_w_* [μg/L] are the sorbed and aqueous concentrations of sorbates at equilibrium, respectively, and *K_F_* [(μg/kg)/(μg/L)^1/n^] and *n* [–] are the Freundlich coefficient and exponent, respectively. Model parameters were obtained using Sigma Plot 12.0 software for Windows.

## Declarations of interest

None.
